# Performance of cytokine models in predicting SLE activity

**DOI:** 10.1186/s13075-019-2029-1

**Published:** 2019-12-16

**Authors:** Nopparat Ruchakorn, Pintip Ngamjanyaporn, Thanitta Suangtamai, Thanuchporn Kafaksom, Charin Polpanumas, Veerachat Petpisit, Trairak Pisitkun, Prapaporn Pisitkun

**Affiliations:** 1 Division of Rheumatology, Department of Medicine, Faculty of Medicine, Srinakharinwirot University Ongkharak Campus, Nakorn Nayok, 26120 Thailand; 2 Division of Allergy, Immunology, and Rheumatology, Department of Medicine, Faculty of Medicine, Ramathibodi Hospital, Mahidol University, Bangkok, 10400 Thailand; 3 BridgeAsia Healthcare Ventures, Bangkok, 10330 Thailand; 4 Center of Excellence in Systems Biology, Faculty of Medicine, Chulalongkorn University, Bangkok, 10330 Thailand; 5 Translational Medicine Program, Faculty of Medicine, Ramathibodi Hospital, Mahidol University, Bangkok, 10400 Thailand

**Keywords:** Cytokines, Disease activity, Systemic lupus erythematosus, Interleukin, Biomarker

## Abstract

**Background:**

Identification of universal biomarkers to predict systemic lupus erythematosus (SLE) flares is challenging due to the heterogeneity of the disease. Several biomarkers have been reported. However, the data of validated biomarkers to use as a predictor for lupus flares show variation. This study aimed to identify the biomarkers that are sensitive and specific to predict lupus flares.

**Methods:**

One hundred and twenty-four SLE patients enrolled in this study and were prospectively followed up. The evaluation of disease activity achieved by the SLE disease activity index (SLEDAI-2K) and clinical SLEDAI (modified SLEDAI). Patients with active SLE were categorized into renal or non-renal flares. Serum cytokines were measured by multiplex bead-based flow cytometry. The correlation and logistic regression analysis were performed.

**Results:**

Levels of IFN-α, MCP-1, IL-6, IL-8, and IL-18 significantly increased in active SLE and correlated with clinical SLEDAI. Complement C3 showed a weakly negative relationship with IFN-α and IL-18. IL-18 showed the highest positive likelihood ratios for active SLE. Multiple logistic regression analysis showed that IL-6, IL-8, and IL-18 significantly increased odds ratio (OR) for active SLE at baseline while complement C3 and IL-18 increased OR for active SLE at 12 weeks. IL-18 and IL-6 yielded higher sensitivity and specificity than anti-dsDNA and C3 to predict active renal and active non-renal, respectively.

**Conclusion:**

The heterogeneity of SLE pathogenesis leads to different signaling mechanisms and mediates through several cytokines. The monitoring of cytokines increases the sensitivity and specificity to determine SLE disease activity. IL-18 predicts the risk of active renal SLE while IL-6 and IL-8 predict the risk of active non-renal. The sensitivity and specificity of these cytokines are higher than the anti-dsDNA or C3. We propose to use the serum level of IL-18, IL-6, and IL-8 to monitor SLE disease activity in clinical practice.

## Background

Systemic lupus erythematosus (SLE) is a chronic autoimmune disease which affects several organ systems including, but not limited to, skin, joint, and renal. The heterogeneity of SLE manifestations arises from the interaction of genetic aberration, immunity, and associated environmental factors [1, 2]. The disturbances of innate and adaptive immunity lead to an increase in inflammatory cytokines, immune complex deposition, autoantibody production, and organ damage. The chronic inflammatory state can lead to morbidity and mortality in SLE patients, e.g., the severe renal flares associated with end-stage renal disease [3]. To decrease the devastating consequence in SLE, the early aggressive treatment of patients who are prone to develop disease flare will improve the long-term outcome.

Cytokines play vital roles in the differentiation, maturation, and activation of many immune lineages. These cytokines influence the gene expression of the immune cells and stimulate the inflammatory process in multiple organs. Several cytokines contribute to SLE pathogenesis [4–6]. Genome-wide association studies (GWAS) have identified several SLE susceptibility genes located in the pathway of type I interferon production and signaling [7]. SLE patients also show the increased expression of interferon-inducible genes in the peripheral blood mononuclear cells (PBMC) [7]. Other cytokines, including interleukin-6 (IL-6), interleukin-10 (IL-10), interleukin-17 (IL-17), and interferon-gamma (IFN-γ), play significant roles in SLE pathogenesis [8–11]. Serum cytokines of IFN- α, IL-6, IL-8, IL-17, and IL-18 correlate with SLE disease severity measured by Systemic Lupus Erythematosus Disease Activity Index (SLEDAI) [12–16]. Circulating IL-18 level is proposed to predict renal damage, which can potentially be a marker to foretell renal involvement and identify patients at risk of kidney failure [17].

The levels of anti-dsDNA and complement, a component in SLEDAI-2 K, have been commonly used to evaluate SLE disease activity [18]. However, these biomarkers do not consistently correlate with clinical SLE flares, especially non-renal manifestation, and there are no standard biomarkers for prediction of SLE flares at the moment [16, 19]. Thus, the monitoring of cytokines involved in lupus pathogenesis may be a beneficial tool to assess clinical SLE activity [4, 20]. Each SLE patient may exhibit the alteration of various cytokines depending on disease severity or type of organ involvement. Recent studies in the subgroup of SLE patients showed the expression of different cytokine profiles, which correlated with disease activity and specific clinical manifestations [21, 22]. Based on these findings, inspecting several cytokines instead of only one cytokine at the same time may increase the yield to predict SLE activity with specific organ involvement.

Our goals of the study were the identification of biomarkers that correlated with SLE disease activity. Once that biomarkers were recognized, we would identify and create a model that showed the best performance in prediction SLE activity. Here we measured the panel of 13 inflammatory cytokines including IL-1β, IFN-α, IFN-γ, TNF-α, MCP-1, IL-6, IL-8, IL-10, IL-12, IL-17, IL-18, IL-23, and IL-33 by multiplex cytokine assays using flow cytometry, verified the sensitivity and specificity, and compared the performance of these cytokines with conventional biomarkers (C3, C4, and anti-dsDNA) to predict SLE flare.

## Methods

### Study population

One-hundred and twenty-four SLE patients who followed up at Rheumatology Clinic during 2016–2017 were enrolled. All of the patients were older than 18 years and met the 1997 ACR criteria [23] or SLICC criteria 2012 [24] for the classification of SLE. The exclusion criteria are SLE patients with overlapping syndrome, a history of cancer, or an active infection. The collected data consisted of demographic information, clinical manifestations, laboratory tests, and treatment history. All patients received standard treatment according to their disease activity and degree of organ damage. The data were collected at the enrollment and up to 3 months of follow-up. The data on patients with active status and inactive status was assessed every 4 weeks, and at the 12th weeks, respectively. The study (ID 07-59-05) was approved by the Faculty of Medicine Ramathibodi Hospital ethics committee and conducted according to the guidelines of the Declaration of Helsinki. Each participant gave written informed consent before enrollment.

Since several cytokines were tested in this study, we selected the data of IL-6 from the paper to calculate the sample sizes [25]. Also, our previous cross-sectional study found that IL-6 is a cytokine that changed in the SLE patients and correlated with disease activity [16]. Sample size estimation for independent *T*-test was calculated with the formulation as follows: *N* = 2σ^2^ (*Z*_1 − *α*/2_ + *Z*_1 − *β*/2_)^2^/*δ*^2^ and *α* = 0.05, *β* = 0.2, *δ* = 58, *σ* = 190. Thus the sample sizes were 33 patients per SLE group (active or inactive). However, we did not know that the other cytokines measuring here will show the difference with the sample sizes calculated here. Thus, we enrolled the patients more (double of sample sizes) to increase the power of the study.

### Measurement of SLE disease activity

SLE clinical disease activity was measured by the Systemic Lupus Erythematosus Disease Activity Index 2000 (SLEDAI-2K) and modified SLEDAI-2K [18], which is a validated tool for measuring disease activity at any given time-point. Physicians evaluated the SLEDAI-2K and modified SLEDAI-2K in each SLE patient visiting a lupus clinic. The clinically active disease was defined if the modified SLEDAI-2K score was ≥ 1, and the disease is clinically inactive if the modified SLEDAI-2K score was 0. The definition of renal active included proteinuria > 0.5 g/day, new or recent onset of proteinuria that increases > 0.5 g/day, cellular casts, red blood cell > 5/HPF, or white blood cells > 5/HPF (exclude infection). The definition of non-renal flare included all clinical parameters in modified SLEDAI with the exclusion of proteinuria, cellular cast, hematuria, and pyuria.

### Measurement of serum cytokines

The sera of SLE patients were collected at the time of enrollment and were used to test for C3, C4, anti-dsDNA, and cytokines. The repeated freeze/thaw cycle was identified to affect the concentration of cytokines [26]. In this study, the stored sera were thawed for the first time and immediately stained with antibodies and subsequently quantified using a multiplex bead-based assay (#740809, LEGENDplex™, Biolegend Inc., USA). The assay can detect 13 of inflammatory cytokines/chemokines, including IL-1β, IFN-α, IFN-γ, TNF-α, MCP-1 (CCL2), IL-6, IL-8 (CXCL8), IL-10, IL-12p70, IL-17A, IL-18, IL-23, and IL-33. The staining procedures were performed as suggested by the manufacturer protocol. The flow cytometry was performed using BD FACSVerse™ (Becton Dickinson Biosciences, San Jose, USA). The flow cytometer raw data files (FCS 2.0, 3.0) were analyzed using the LEGENDplexTM Data Analysis Software. The signal value was detected on LEGENDplex chip platform, and the serum concentration was calculated according to the standard curve. The concentration range was 0–30,000 pg/ml for all 13 cytokines, and the sensitivity of detection achieved 0.01 pg/ml level. The coefficient of variation (CV) of the standard testing cross three batches of 13 cytokines showed the median (range) between 2.2 (1.19–4.21), 1.34 (0.79–4.91), and 2.67 (2.31–3.5). The cytokine data were analyzed at weeks 0, 4, 8, and 12 weeks for active SLE patients, and at 0 and 12 weeks for inactive SLE patients.

### Statistical analysis

Statistical analysis was performed using STATA Statistics Data Analysis 14.0 computer software. The mean ± SD was used to describe normally distributed data, median and range for skewed data, and the percentage was used to represent categorical data. Mann-Whitney *U* test was used to compare the median from two groups if skewed distribution existed. Pearson’s correlation analyzed the correlations between serum cytokines and SLEDAI scores and showed a significant level (*p* value). The Bonferroni correction was performed to adjust the *p* value for multiple comparisons. Receiver operating characteristic (ROC) curves discriminated active from inactive SLE for each of serum cytokines, anti-dsDNA, C3, and C4. Logistic regression models were used to predict active SLE status and lupus nephritis. The results were considered statistically significant if the *p* value was < 0.05. The power of 0.8 was used to calculate the sample size for the primary outcome.

## Results

### Clinical characteristics of the study population

One-hundred and twenty-four patients participated in this study. Patients were categorized into active or inactive SLE based on the modified SLEDAI-2K. Of the total 124 patients, 51 cases (41%) have active SLE, whereas 73 cases (59%) have inactive SLE. Active and inactive SLE group had a median disease duration of 63.63 and 102.90 months, respectively. The median of clinical SLEDAI score at the baseline in the active group was 8 (Table 1). A major difference in clinical manifestation between active and inactive SLE was renal involvement. Thirty-one out of 51 patients (60%) in the active group showed symptoms and signs of lupus nephritis. The patients received different immunosuppressive agents, as indicated (Additional file 1: Table S1). The active SLE patients significantly received a higher dose of prednisolone and more usage of cyclophosphamide than the inactive SLE patients (Additional file 1: Table S1).

**Table 1:** Demographics and clinical characteristics of patients

Parameter	Active SLE (*n* = 51)	Inactive SLE (*n* = 73)	*p* value
Age, year (mean ± SD)	35.79 ± 14.31	41.01 ± 12.71	1.000
Gender, male, *n* (%)	2 (3.92)	4 (5.48)	1.000
Female, *n* (%)	49 (96.08)	69 (94.52)	
BMI, kg/m^2^, (#)	21.25 (14.67–30.85)	23.06 (15.89–46.70)	1.000
Age onset, year (mean ± SD)	29.55 ± 14.25	31.74 ± 12.50	1.000
Disease duration, month, (#)	63.63 (0–383.40)	102.90 (4.40–482.10)	1.000
Duration of last active, month, (#) ***	2.50 (0–13.00)	8.20 (0.90–165.10)	< 0.001
Hypertension, *n* (%)	6 (11.76)	9 (12.33)	1.000
Diabetes mellitus, *n* (%)	4 (7.84)	5 (6.85)	1.000
Clinical SLEDAI score, (#) ***	8 (1–36)	0	< 0.001
ESR, mm/h (#)	33 (5–120)	22 (5–98)	0.519
ESR < 20 mm/h, *n* (%)	15 (29.4)	34 (46.6)	1.000
ESR > 20 mm/h, *n* (%)	36 (70.6)	39 (53.4)	1.000
WBC, cells/mm^3^ (#)	6220 (2700-17,290)	5500 (3060-13,940)	1.000
Hemoglobin, g/dl (#) *	12.00 (6.70–14.00)	12.00 (9.30–15.60)	0.034
Platelet, ×103 cells/mm^3^ (#)	271 (57–458)	262 (127–445)	1.000
Serum creatinine, mg/dl (#)	0.80 (0.40–1.89)	0.70 (0.50–1.28)	1.000
UPCR, (#)	1.71 (0.12–33.33)	0.12 (0.03–0.40)	0.534
UPCR < 0.5, *n* (%) ***	22 (43.1)	73 (100)	< 0.001
UPCR > 0.5, *n* (%) ***	29 (56.9)	0 (0)	< 0.001
UPCR > 1.0, *n* (%) ***	20 (39.2)	0 (0)	< 0.001

*BMI* body mass index*, ESR* erythrocyte sedimentation rate, *UPCR* urine protein to creatinine ratio

# = [median (range)]; *p <  0.05, ***p <  0.001 (Bonferroni correction)

### Correlation of serum biomarkers and clinical SLEDAI score

SLE patients (both active and inactive) showed the difference in serum cytokine levels (IFN-α, MCP-1, IL-8, and IL-18) compared to normal controls at time 0 (Additional file 2: Table S2). Among all cytokines tested, the levels of MCP-1, IL-6, IL-8, and IL-18 were significantly higher in the active SLE patients compared with those in the inactive SLE (Table 2). Next, we determined the difference and the correlation of serum biomarkers between active and inactive SLE at time 0. We analyzed the correlation of these biomarkers with SLE clinical activity to show the correlation coefficient (R). The serum biomarkers that provided substantial correlation coefficient with clinical SLEDAI scores were C3, IFN-α, IL-6, IL-8, and IL-18 (Table 2). Also, IL-8, IL-18, and IL-6 yielded a higher correlation coefficient than standard biomarkers (C3, C4, and anti-dsDNA) as shown (Table 2). The graph demonstrated the correlation between these biomarkers and modified SLEDAI was shown (Additional file 6: Figure S1).

**Table 2:** Correlation of serum biomarkers and SLE disease activity

Biomarker	Active (*n* = 51) [median (range)]	Inactive (*n* = 73) [median (range)]	*p* value^A^	*R*	*p* value^B^
C3, g/l	0.84 (0.18–1.80)	0.95 (0.52–1.63)	0.080	−0.406	< 0.001***
C4, g/l	0.17 (0.01–0.69)	0.22 (0.03–0.47)	0.528	−0.203	1.000
Anti-dsDNA, IU/ml	64.30 (10.0–800.0)	24.75 (10.0–800.0)	0.352	0.223	1.000
IL-1β, pg/ml	1.39 (1.10–33.97)	1.39 (1.25–1.39)	1.000	0.126	1.000
IFN-α, pg/ml	8.51 (1.48–449.29)	1.54 (1.48–89.25)	0.272	0.374	0.002**
IFN-γ, pg/ml	1.52 (1.52–966.81)	1.58 (1.52–47.24)	1.000	0.126	0.223
TNF-α, pg/ml	2.22 (1.90–11.34)	2.22 (1.22–56.74)	1.000	−0.047	1.000
MCP-1, pg/ml	1111.96 (330.92–20,499.66)	609.42 (4.54–14,597.43)	0.016*	0.211	1.000
IL-6, pg/ml	22.73 (1.27–608.74)	1.87 (1.00–155.65)	< 0.001***	0.450	< 0.001***
IL-8, pg/ml	70.77 (1.28–840.89)	16.78 (1.13–220.54)	< 0.001***	0.541	< 0.001***
IL-10, pg/ml	8.96 (1.22–482.23)	1.42 (1.22–49.32)	1.000	0.103	0.101
IL-12, pg/ml	1.06 (1.06–10.61)	1.06 (1.06–4.52)	1.000	0.108	1.000
IL-17, pg/ml	6.34 (1.18–61.96)	1.47 (1.00–432.93)	1.000	0.062	1.000
IL-18, pg/ml	177.87 (1.14–2526.77)	7.36 (1.14–1288.23)	< 0.001***	0.462	< 0.001***
IL-23, pg/ml	2.52 (2.03–88.94)	2.52 (2.03–76.62)	1.000	0.046	1.000
IL-33, pg/ml	1.66 (0.88–32.29)	1.66 (1.01–2.19)	1.000	−0.009	1.000

A = Mann-Whitney *U* test, R = correlation coefficient, B = Pearson’s correlation; *p <  0.05, **p < 0.01, ***p < 0.001 (Bonferroni correction)

Furthermore, we analyzed the correlation between cytokines and the immunosuppressive drugs that patients received, which might suppress the level of cytokines (Additional file 3: Table S3). We did not observe the association between immunosuppressive drugs and cytokines. However, the dosage of prednisolone correlated positively with IL-8 and IL-18. The data suggested that the active SLE had higher levels of IL-8 and IL-18, and concurrently received a higher dose of prednisolone to control disease activity. Also, the factors that may affect the cytokine levels are age and gender. We analyzed the correlation between cytokines and these factors (Additional file 4: Table S4). Our data showed a negligible correlation (*R* <  0.2) between cytokines, age, and gender in SLE patients. The analyses suggested that the cytokine levels of SLE patients in this study most likely were modulated by clinical disease activity.

### Association of serum biomarkers in SLE patients

The previous study showed that serum IL-6 correlates with modified SLEDAI but not anti-dsDNA, complement, and SLEDAI-2K [16]. We analyzed the correlation among cytokines that correlated with clinically active SLE and standard biomarkers (complement and anti-dsDNA). C3 correlated with only IFN-α while anti-dsDNA associated with IFN-α and MCP-1 (Table 3). The correlation analysis of IFN-α, MCP-1, IL-6, IL-8, and IL-18 showed significantly correlated among these cytokines to a certain degree (Table 3). The graphs of the correlation between these biomarkers were shown (Additional file 7: Figure S2). The data suggested that most of cytokines that correlated with SLE disease activity did not correlate with standard biomarkers. The combination of biomarkers may provide better sensitivity and specificity to predict SLE disease activity.

**Table 3:** The correlation of serum biomarkers in all SLE patients (*N* = 124)

Biomarkers		C3	C4	Anti-DNA	IFN-α	MCP-1	IL-6	IL-8	IL-18
C3	R	–	0.718	−0.324	− 0.363	−0.122	− 0.214	−0.242	− 0.130
	P	–	***	**	***	ns	ns	ns	ns
C4	R	0.718	–	−0.220	−0.244	− 0039	**−** 0.113	−0.157	− 0.051
	P	***	–	ns	ns	ns	ns	ns	ns
Anti-dsDNA	R	−0.324	−0.220	–	0.323	0.292	0.125	0.201	0.177
	P	**	ns	–	**	*	ns	ns	ns
IFN-α	R	−0.363	−0.244	0.323	–	0.450	0.653	0.721	0.602
	P	***	ns	**	–	***	***	***	***
MCP-1	R	−0.122	−0039	0.292	0.450	–	0.330	0.470	0.412
	P	ns	ns	*	***	–	**	***	***
IL-6	R	−0.214	**−**0.113	0.125	0.653	0.330	–	0.784	0.712
	P	ns	ns	ns	***	**	–	***	***
IL-8	R	**−**0.242	−0.157	0.201	0.721	0.470	0.784	–	0.720
	P	ns	ns	ns	***	***	***	–	***
IL-18	R	−0.130	−0.051	0.177	0.602	0.412	0.712	0.720	–
	P	ns	ns	ns	***	***	***	***	–

R = correlation coefficient; *p < 0.05, **p < 0.01, p*** < 0.001, *ns* non-significant (Bonferroni correction)

### Discrimination between active and inactive SLE by serum biomarkers

Although C3, C4, and anti-dsDNA are used as conventional biomarkers to monitor SLE disease activity and are the components in SLEDAI-2K, the sensitivity of these traditional biomarkers are still limited [27, 28]. The receiver operator characteristic (ROC) curves were portrayed to determine whether the selected biomarkers that correlated with clinical SLEDAI can predict the active and inactive clinical symptoms of SLE patients. The ROC curve analysis suggested that IL-8, IL-18, IL-6, IFN-α, and MCP-1 are sufficient to discriminate between active or inactive SLE (based on clinical SLEDAI score). Analysis of area under the curve (AUC) showed IL-18, IL-8, IL-6, and MCP-1 gave the higher AUC than C3, C4, and anti-dsDNA (Table 4).

**Table 4:** Performance of biomarkers to predict active SLE

Biomarker	AUC	95% CI	Cut-off point	Sensitivity	Specificity	LR+	LR−
C3	0.647	0.541–0.752	< 0.90 g/l	61.64%	71.23%	1.57	0.63
C4	0.612	0.500–0.724	< 0.20 g/l	47.06%	63.10%	1.48	0.68
Anti-dsDNA	0.619	0.519–0.719	> 100.0 IU/ml	52.94%	63.01%	1.43	0.74
IFN-α	0.622	0.523–0.720	> 10.17 pg/ml	50.98%	59.80%	1.68	0.70
MCP-1	0.707	0.616–0.799	> 779.28 pg/ml	66.67%	67.12%	2.02	0.49
IL-6	0.684	0.592–0.776	> 5.44 pg/ml	52.94%	82.19%	2.97	0.57
IL-8	0.754	0.663–0.844	> 55.0 pg/ml	64.00%	82.43%	3.38	0.44
IL-18	0.801	0.721–0.882	> 50.0 pg/ml	70.00%	79.73%	3.45	0.37

*AUC* area under a receiver operating characteristic (ROC) curve, *LR* likelihood ratios

Number of patients using in the analysis; active SLE (*n* = 51), inactive SLE (*n* = 73)

Next, we selected the cut-off points of these biomarkers from ROC curves that yielded the highest sensitivity and specificity to predict SLE disease activity (both renal and non-renal). The ROC curves of these biomarkers were graphed and demonstrated (Additional file 8: Figure S3). Serum IL-18 had the highest AUC (0.801), and the cut-off at 50 pg/ml showed 70% sensitivity and 79.73% specificity (Table 4). The positive Likelihood Ratios (LR+) of IL-8, IL-18, IL-6, IFN-α, and MCP-1 are higher than the complement and anti-dsDNA (Table 4).

### Predictors of SLE disease activity during follow-up by logistic regression analysis

SLE patients showed various clinical flares that may associate with different types of cytokine-mediated in the pathogenesis. We characterized the group of clinical SLE into three groups, i.e., active any clinical (active SLE), active renal, and active non-renal. Next, we analyzed the candidate biomarkers to use as a predictor of SLE disease flares and chose the primary variables from bivariate analyses that showed significant *p* value (< 0.05) for further analysis with the multiple logistic regression. Bivariate analyses yielded statistically significant OR for active SLE at the baseline by the following cytokines: IL-18, IL-8, IL-6, IFN-α, MCP-1, and C3 for active SLE (Table 5). Next, we selected the biomarkers that yielded the significant OR from bivariate logistic regression and analyzed OR using multiple regression analysis which showed that only three cytokines (IL-8, IL-18, and IL-6) that still had statistically significant OR for active SLE status at the baseline (Table 5). IL-18 and IL-6 increased OR for active renal and active non-renal, respectively (Table 5).

**Table 5:** Predictors of SLE disease activity by logistic regression analysis

Biomarkers	Weeks	Model	Active SLE (*N* = 51)		Active renal (*N* = 31)		Active non-renal (*N* = 20)	
			ORs (95%CI)	*p* value	ORs (95%CI)	*p* value	ORs (95%CI)	*p* value
C3 (< 0.9 g/l)	0	Bivariate	2.22 (1.10–4.61)	0.032	7.90 (2.52–24.74)	< 0.001	2.12 (0.80–5.64)	0.129
		Multiple	2.33 (0.98–53.5)	0.062	4.63 (1.48–14.44)	0.008	–	–
	12	Bivariate	2.52 (1.71–3.69)	< 0.001	2.82 (1.82–4.37)	< 0.001	1.25 (0.75–2.07)	0.383
		Multiple	2.54 (1.46–4.42)	0.001	3.27 (1.69–6.32)	< 0.001	–	–
C4 (< 0.2 g/l)	0	Bivariate	1.14 (0.53–2.44)	0.738	7.91 (2.38–26.33)	0.001	2.01 (0.75–5.36)	0.159
		Multiple	–	–	0.82 (0.24–2.78)	0.759	–	–
	12	Bivariate	2.16 (1.47–3.18)	< 0.001	2.27 (1.47–3.50)	< 0.001	1..27 (0.76–2.11)	0.354
		Multiple	1.22 (0.70–2.12)	0.465	1.08 (0.57–2.07)	0.793	–	–
Anti-dsDNA (> 100.0 IU/ml)	0	Bivariate	1.87 (0.88–3.98)	0.100	1.21 (0.43–3.36)	0.714	1.01 (0.37–2.77)	0.974
		Multiple	–	–	–	–	–	–
	12	Bivariate	1.10 (0.76–1.59)	0.606	1.53 (0.99–2.35)	0.053	0.66 (0.40–1.10)	0.116
		Multiple	–	–	–	–	–	–
IFN-α (> 10.0 pg/ml)	0	Bivariate	2.23 (1.06–4.68)	0.034	3.33 (1.13–9.78)	0.028	1.11 (0.41–2.95)	0.833
		Multiple	0.86 (0.31–2.33)	0.772	1.39 (0.52–3.69)	0.503	–	–
	12	Bivariate	1.01 (0.69–1.46)	0.971	0.99 (0.64–1.51)	0.960	1.03 (0.62–1.70)	0.915
		Multiple	–	–	–	–	–	–
MCP-1 (> 960.0 pg/ml)	0	Bivariate	4.08 (1.90–8.73)	< 0.001	2.09 (0.75–5.84)	0.157	3.18 (1.13–8.93)	0.028
		Multiple	1.07 (0.36–3.21)	0.893	–	–	1.52 (0.43–5.39)	0.510
	12	Bivariate	0.810 (0.56–1.18)	0.269	0.67 (0.44–1.03)	0.072	1.17 (0.71–1.95)	0.535
		Multiple	–	–	–	–	–	–
IL-6 (> 6.2 pg/ml)	0	Bivariate	5.68 (2.26–14.30)	< 0.001	1.56 (0.50–4.85)	0.439	4.47 (1.63–12.25)	0.004
		Multiple	3.49 (1.19–19.1)	0.022	–	–	3.11 (1.05–9.26)	0.041
	12	Bivariate	0.72 (0.50–1.05)	0.089	0.77 (0.50–1.19)	0.252	0.78 (0.47–1.30)	0.340
		Multiple	–	–	–	–	–	–
IL-8 (> 55.0 pg/ml)	0	Bivariate	7.20 (3.22–16.08)	< 0.001	3.62 (1.25–10.44)	0.017	3.09 (1.13–8.42)	0.027
		Multiple	4.86 (1.87–12.57)	0.001	2.50 (0.87–7.15)	0.086	1.33 (0.36–4.84)	0.657
	12	Bivariate	1.12 (0.77–1.65)	0.567	0.78 (0.50–1.20)	0.260	1.81 (1.04–3.17)	0.035
		Multiple	–	–	–	–	1.81 (1.04–3.17)	0.035
IL-18 (> 50.0 pg/ml)	0	Bivariate	9.93 (4.29–22.95)	< 0.001	7.19 (2.25–22.95)	0.001	3.31 (1.17–9.29)	0.023
		Multiple	5.54 (12.2–14.43)	< 0.001	5.37 (1.74–16.57)	0.003	1.86 (0.55–6.32)	0.318
	12	Bivariate	1.55 (1.06–2.27)	0.023	1.90 (1.20–3.00)	0.006	0.94 (0.57–1.55)	0.802
		Multiple	1.96 (1.30–2.94)	0.001	2.53 (1.56–4.14)	< 0.001	–	–

Furthermore, we analyzed whether these biomarkers can predict the disease flare at 12 weeks, the data showed only IL-18 and C3 showed significantly OR to predict active SLE and active renal at 12 weeks (Table 5). Besides, IL-8 showed marginal but significant OR to predict non-renal flare at 12 weeks (Table 5). Next, we analyzed the patients at 4 and 8 weeks to examine if the cytokines could predict the flares at an earlier time (Additional file 5). We also obtained findings similar to the analysis at 12 weeks. C3 and IL-18 predicted future renal flares while IL-8 predicted future non-renal flares. IL-6 predicted non-renal flares at time 0 but did not predict future non-renal flares.

### Sensitivity and specificity of biomarker models to predict SLE disease activity

Moreover, we analyzed the sensitivity, specificity, and likelihood ratio of the possible model of biomarkers to make the judgment for using these parameters in the clinical practice (Table 6). We selected the cytokines to create a model based on the significant data by multiple logistic regression analysis (Table 5). We compared the sensitivity and specificity of the conventional biomarkers and cytokines for the prediction of SLE disease activity. IL-18 showed higher sensitivity and specificity than a model of anti-dsDNA or C3 (standard model) to predict active SLE and active renal (Table 6). A model of IL-8 or IL-18 increased the specificity to predict active SLE at baseline. Also, IL-6 and IL-8 yielded higher sensitivity and specificity than a standard model to predict non-renal active at baseline and 12 weeks, respectively (Table 6).

**Table 6:** Sensitivity and specificity of models to predict SLE disease activity

Disease status	Duration (weeks)	Models	Sensitivity	Specificity	LR+	LR−
Active SLE	0	Anti-dsDNA or C3	61.64%	71.23%	1.57	0.63
		C3	61.64%	71.23%	1.57	0.63
		IL-6	52.94%	82.19%	2.97	0.57
		IL-8	64.00%	82.43%	3.38	0.44
		IL-18	70.00%	79.73%	3.45	0.37
		C3 or IL-18	70.00%	79.73%	3.45	0.37
		IL-8 or IL-18	70.00%	82.43%	3.45	0.44
	12	Anti-dsDNA or C3	59.35%	65.64%	1.46	0.77
		C3	59.35%	59.49%	1.46	0.68
		IL-6	44.84%	65.13%	1.36	0.88
		IL-8	58.71%	57.95%	1.33	0.73
		IL-18	68.39%	67.18%	1.99	0.51
		C3 or IL-18	68.39%	67.18%	1.99	0.51
		IL-8 or IL-18	68.39%	67.18%	1.99	0.51
Active renal	0	Anti-dsDNA or C3	70.96%	68.82%	2.06	0.75
		C3	70.96%	64.51%	2.06	0.44
		IL-18	80.65%	74.19%	3.00	0.26
		C3 or IL-18	80.65%	74.19%	3.00	0.26
	12	Anti-dsDNA or C3	69.57%	65.12%	1.67	0.64
		C3	69.57%	58.52%	1.67	0.52
		IL-18	70.65%	66.28%	1.99	0.45
		C3 or IL-18	70.65%	66.28%	1.99	0.45
Active non-renal	0	Anti-dsDNA or C3	40.00%	64.42%	1.22	0.97
		C3	40.00%	50.96%	1.22	0.78
		IL-6	60.00%	73.08%	2.22	0.54
		IL-8	60.00%	67.31%	1.83	0.59
		C3 or IL-6	60.00%	73.08%	2.22	0.54
		C3 or IL-8	60.00%	67.31%	1.83	0.59
		IL-6 or IL-8	60.00%	73.08%	2.22	0.54
	12	Anti-dsDNA or C3	44.43%	58.89%	1.09	0.97
		C3	44.43%	50.17%	1.09	0.90
		IL-6	33.33%	65.16%	1.02	0.95
		IL-8	65.28%	61.67%	1.51	0.55
		C3 or IL-6	44.43%	65.16%	1.09	0.95
		C3 or IL-8	65.28%	61.67%	1.51	0.55
		IL-6 or IL-8	65.28%	65.16%	1.51	0.95

## Discussion

Autoantibody production from autoreactive cells is a hallmark of SLE and precedes before clinical onset [29]. The presence of autoantibody alone cannot initiate the SLE symptoms unless receiving the second unknown trigger [29]. Cytokine dysregulation can potentiate the loss of immune tolerance resulting in active clinical SLE and end-organ damage [5]. Previous studies showed that serum complement and anti-dsDNA do not always correlate with active SLE, especially non-renal involvement [16, 30]. The serum level of interleukin-6 does not associate with complement and anti-dsDNA but associates with the modified SLEDAI-2K [16]. Thus, we chose the modified SLEDAI-2K instead of SLEDAI-2K, which includes anti-dsDNA and complement, to classify the patients into active and inactive SLE and analyzed the correlation with the inflammatory cytokines. However, not all of the clinical manifestations of SLE are included in SLEDAI. Although several tools are available for clinical evaluation of SLE patients, our study selected modified SLEDAI to determine disease activity according to the user-friendly tool for the clinicians in routine practice. Thus, our study may not capture some clinical manifestations of SLE that do not include in the modified SLEDAI.

Serum cytokines, including IL-8, IL-18, IL-6, IFN-α, and MCP-1 were significantly higher in the active SLE patients than in the inactive ones. These cytokines have been shown the association with SLE disease activity [16, 31–33]. Also, IL-18 is a cytokine that can be detected in active clinical SLE with anti-dsDNA, hypocomplementemia, and arthritis [22]. However, the head-to-head comparisons of performance between these cytokines and conventional biomarkers (complement and anti-dsDNA) to predict the active clinical SLE have never been described. Our data showed that IL-6, IL-8, and IL-18 presented higher correlation coefficients with SLE disease activity than complement and anti-dsDNA. IL-18 yielded the highest sensitivity, while IL-8 gave the highest specificity to predict active SLE. Our data suggested that these inflammatory cytokines increase the sensitivity and specificity to predict SLE disease activity.

The previous study reported that the levels of IL-18 did not differ in SLE patients regardless of glucocorticoid usage. However, that study did not analyze the correlation of glucocorticoid dosage and IL-18 level [34]. The dosage of prednisolone correlated positively with levels of IL-18 in this study. The active patients had a high level of IL-18 and concurrently received a higher dose of a glucocorticoid to control the disease activity. The inactive patients had a low level of IL-18 and received a low dose of glucocorticoid. These three variables (disease activity, IL-18, and steroid dosage) correlated in a positive direction. However, the steroid can reduce IL-18 production in stimulated healthy PBMC [35]. These findings suggested that glucocorticoid was prescribed to control active SLE disease while IL-18 increased with SLE disease activity.

One of the difficult tasks during follow-up SLE patients is to control the disease activity and maintain a remission state. The biomarkers that can predict the disease flare at the next follow-up to guide the clinicians to adjust the medication would be very useful. Serum complements (C3 and C4) correlate with lupus nephritis, but they do not associate with total disease activity as well as SLE non-renal activity [27, 28]. The cytokines involved in the pathogenesis of SLE associated with non-renal disease activity [36]. Thus, we characterized the active clinical SLE into renal or non-renal manifestations and analyzed by several statistical methods, including correlation analysis with multiple testing correction, OR, sensitivity, specificity, and multiple-regression analysis. The sensitivities and specificities were reported here based on the discovery dataset, they may probably overestimate of the true value (inevitable overfitting) and that therefore this work is hypothesis-generating: a validation cohort is required to understand the true performance of the cytokines identified. Our data showed two subsets of cytokines that predicted renal and non-renal activity.

IL-6 and IL-18 increased the OR of active non-renal and renal flares, respectively. This result confirmed the previous works that showed the IL-6 is a sensitive marker for active non-renal SLE [16, 36]. The plasma concentration of IL-18 correlates with renal involvement in SLE [37]. The complement C3 level tends to be higher in the milder lupus nephritis than the severe proliferative nephritis [36]. Our patients in this cohort were followed up at the outpatient clinic, in which the severity of renal activity might not be very critical. However, we did see the significant OR of C3 to predict active renal SLE in this cohort. Also, IL-18 levels did change and show the significant OR to predict active renal SLE at time 0. The OR of IL-18 was higher than C3, which suggested the performance of IL-18 better than the complement as a biomarker for active renal SLE.

Furthermore, the analysis of data at 4, 8, and 12 weeks showed that C3 and IL-18 had significant OR to predict future renal active. Surprisingly, we found only IL-8 showed slightly increased OR of non-renal active at 4, 8, and 12 weeks. IL-8 can be used as a marker to predict future non-renal flares. IL-8 has been detected in the cerebrospinal fluid of patients with central neuropsychiatric lupus erythematosus [38]. Nucleosome activation in polymorphonuclear cells (PMN) can increase the secretion of IL-8, the chemoattractant for neutrophil recruitment [39]. The IL-8 initiates the neutrophil extracellular traps (NETs) which activate type I IFN production [40]. SLE patients have high levels of IL-8 together with the defect to degrade NETs resulting in the aggravation of the inflammatory processes [31, 41]. The rise of IL-8 may be the initial activation of the inflammatory process leading to clinical SLE flares and that is the reason why IL-8 can be used as a predictor for future SLE non-renal flare.

Our study conducted in patients with active disease and free of infection at the time of cytokine analysis. This study demonstrated the cytokine changed in the patients were mainly affected by SLE, and not because of the infection. One of the most difficult tasks for treating lupus patients is to differentiate the clinical manifestation between active disease and infection. The study of cytokines in SLE patients at risk to develop infection may demonstrate that the different patterns of cytokines changed, which will be a useful marker to differentiate between active SLE and SLE with infection. A longitudinal study is required to prove this concept. Although our study was conducted in a tertiary hospital in which patients were referred from several major regions in the country, our data showed common findings with the previous study; the correlation of IL-18 with renal disease activity and IL-6 with the non-renal disease have been described. Thus, we consider that our findings are global and can apply to SLE patients in other ethnic groups.

In brief, all of the analyses directed to the three cytokines (IL-6, IL-8, and IL-18) that predicted SLE disease activity with good sensitivity and specificity. IL-6 predicted non-renal flare at time 0 while IL-8 predicted future non-renal flare. IL-18 predicted renal flare at time 0 and future flare. The parameters that will help to select the cytokines to predict disease activity are urine protein and sediments: renal active, select IL-18, and non-renal active, select IL-6, and IL-8. A rapid diagnostic test to measure these cytokines would be a useful tool to monitor SLE disease activity.

## Conclusion

Two sets of cytokines should be monitored to predict renal and non-renal flares in SLE. IL-18 predicted renal flare while IL-6 and IL-8 predicted non-renal flare. The performance of these cytokines to predict active clinical SLE is superior to complement and anti-dsDNA. IL-18, IL-8, and IL-6 should be used in addition to conventional biomarkers to increase the sensitivity and specificity to determine SLE activity.

## Supplementary information

**Additional file 1: Table S1.** Medications in SLE patients in this cohort.

**Additional file 2: Table S2.** Cytokines in normal controls and SLE patients.

**Additional file 3: Table S3.** The correlation between cytokines and medications in all SLE patients.

**Additional file 4: Table S4.** The correlation between cytokines, age, and gender in SLE patients.

**Additional file 5: Table S5.** Predictors of SLE disease activity by logistic regression analysis.

**Additional file 6: Figure S1.** The correlation of biomarkers and modified SLEDAI in SLE patients.

**Additional file 7: Figure S2.** The correlation between each cytokine in all SLE patients.

**Additional file 8: Figure S3.** Receiver operating characteristic (ROC) curve and area under the ROC curve in SLE patients.

## Data Availability

Data and material available for this study would require further approval upon request from the corresponding author.

## Abbreviations

ACR: American College of Rheumatology
Anti-dsDNA: Anti-double stranded DNA
AUC: Area under the curve
BMI: Body mass index
CCL: Chemokine ligand
CXCL: CXC chemokine ligand
ESR: Erythrocyte sedimentation rate
GFR: Glomerular filtration rate
GWAS: Genome-wide association studies
IFN: Interferon
IL: Interleukin
LR: Likelihood ratios
MCP: Monocyte chemoattractant protein
MD: Medical doctor
MS: Master of science
NETs: Neutrophil extracellular traps
Ns: Non-significant
OR: Odds ratio
PBMC: Peripheral blood mononuclear cells
PMN: Polymorphonuclear cells
R: Correlation coefficient
ROC: Receiver operating characteristic
SD: Standard deviations
SLE: Systemic lupus erythematosus
SLEDAI-2K: SLE disease activity index-2K
SLICC: Systemic lupus erythematosus international collaborating clinics
TNF: Tumor necrosis factor
UPCR: Urine protein to creatinine ratio
WBC: White blood cell
α: Alpha
β: Beta
γ: Gamma
