# Supplementary material for: Performance of cytokine models in predicting SLE activity
Source: Arthritis Res Ther. 2019 Dec 16;21:287. doi: 10.1186/s13075-019-2029-1 (PMC6915901; doi:10.1186/s13075-019-2029-1)
Supplement: Supplementary file 1 — Additional file 1: Table S1. Medications in SLE patients in this cohort. [file 13075_2019_2029_MOESM1_ESM.docx]

**Table S1*.* Medications in the SLE patients in this cohort**

| **Medications** | **Total**  **(n=124)** | **Active SLE**  **(n=51)** | **Inactive SLE**  **(n=73)** | **P-value** |
| --- | --- | --- | --- | --- |
| **Prednisolone** |  |  |  |  |
| Current use, n (%) | 106(85.48) | 51(100) | 55(75.34) | 0.062 |
| Current daily dose, mg  (mean±SD) ******* | 11.96±12.68 | 23.06±12.87 | 4.20±3.37 | <0.001 |
| **Hydroxychloroquine** |  |  |  |  |
| Current use, n (%) | 99(79.83) | 43(84.31) | 56(76.71) | 1.000 |
| Current daily dose, mg  (mean±SD) | 185.67±66.56 | 202.45±53.15 | 172.78±73.13 | 1.000 |
| **Azathioprine** |  |  |  |  |
| Current use, n (%) | 54(43.54) | 14(27.45) | 40(54.79) | 0.312 |
| Current daily dose, mg  (mean±SD) | 64.34±24.79 | 63.26±31.30 | 64.72±22.54 | 1.000 |
| **Cyclophosphamide** |  |  |  |  |
| Current use, n (%) ******* | 36(29.03) | 30(58.82) | 6(8.22) | <0.001 |
| Current daily dose, mg  (mean±SD) | 35.60±12.09 | 34.63±9.29 | 40.46±22.11 | 1.000 |
| **Mycophenolate mofetil** |  |  |  |  |
| Current use, n (%) | 38(30.64) | 18(35.29) | 20(27.40) | 1.000 |
| Current daily dose, mg  (mean±SD) | 1,423.68±590.77 | 1,638.88±613.70 | 1,230.00±509.23 | 1.000 |
| **Tacrolimus** |  |  |  |  |
| Current use, n (%) | 8(6.45) | 7(13.73) | 2(2.74) | 1.000 |
| Current daily dose, mg  (mean±SD) | 3.05±1.42 | 3.21±1.57 | 2.50±0.70 | 1.000 |
| **Cyclosporin A** |  |  |  |  |
| Current use, n (%) | 5(4.03) | 1(1.96) | 4(5.48) | 1.000 |
| Current daily dose, mg  (mean±SD) | 105±51.23 | 25 | 125±28.86 | 1.000 |
| **Methotrexate** |  |  |  |  |
| Current use, n (%) | 17(13.70) | 5(9.80) | 12(16.44) | 1.000 |
| Current daily dose, mg  (mean±SD) | 1.81±0.66 | 2.1 | 1.69±0.76 | 1.000 |

*p<0.05, **p<0.01, ***p<0.001 (Bonferroni correction)
