# Supplementary material for: Performance of cytokine models in predicting SLE activity
Source: Arthritis Res Ther. 2019 Dec 16;21:287. doi: 10.1186/s13075-019-2029-1 (PMC6915901; doi:10.1186/s13075-019-2029-1)
Supplement: Supplementary file 2 — Additional file 2: Table S2. Cytokines in normal controls and SLE patients. [file 13075_2019_2029_MOESM2_ESM.docx]

**Table S2*.* Cytokines in normal controls and SLE patients**

| **Cytokines**  **(pg/ml)** | **Normal**  **controls**  **(n =10)** | **SLE patients** | | |
| --- | --- | --- | --- | --- |
|  |  | **Inactive (n=73)** | **Active (n=51)** | **Total (n=124)** |
| IL-1β | 1.48  (0-1.48) | 1.39  (1.25-1.39) | 1.39  (1.10-33.97) | 1.64  (1.19-1.48) |
| IFN-α | 1.54  (1.54-8.52) | 1.54*  (1.48-89.25) | 8.51*  (1.48-449.29) | 2.79*  (1.48-271.67) |
| IFN-γ | 1.52  (1.52-3.22) | 1.58  (1.52-47.24) | 1.52  (1.52-966.81) | 1.52  (1.52-217.07) |
| TNF-α | 2.13  (0-2.13) | 2.22  (1.22-56.74) | 2.22  (1.90-11.34) | 2.22  (1.22-11.86) |
| MCP-1 | 296.07  (107.30-685.18) | 609.42*  (4.54-14,597.43) | 1,111.96*  (330.92-20,499.66) | 687.36*  (82.09-18,414.60) |
| IL-6 | 1.86  (0-1.86) | 1.87  (1.00-155.65) | 22.73*  (1.27-608.74) | 4.75  (1.00-242.01) |
| IL-8 | 15.05  (2.10-32.17) | 16.78*  (1.13-220.54) | 70.77*  (1.28-840.89) | 34.85*  (1.13-686.56) |
| IL-10 | 1.20  (1.22-9.65) | 1.42  (1.22-49.32) | 8.96  (1.22-482.23) | 1.84  (1.22-171.25) |
| IL-12 | 1.21  (0-1.21) | 1.06  (1.06-4.52) | 1.06  (1.06-10.61) | 1.06  (1.06-4.52) |
| IL-17 | 1.65  (0-1.65) | 1.47  (1.00-432.93 ) | 6.34*  (1.18-61.96) | 1.47  (1.00-199.65) |
| IL-18 | 1.03  (1.03-8.67) | 7.36*  (1.14-1,288.23) | 177.87*  (1.14-2,526.77) | 173.82*  (1.07-1980.86) |
| IL-23 | 1.22  (0-1.22) | 2.52  (2.03-76.62) | 2.52  (2.03-88.94) | 2.52  (2.03-76.62) |
| IL-33 | 1.15  (0-1.15) | 1.66  (1.01-2.19) | 1.66  (0.88-32.29) | 1.66  (1.01-2.19) |

Data show in median (range); p-value; compared with normal controls, *p<0.05, **p<0.01, ***p<0.001 (Bonferroni correction)
