# Supplementary material for: Performance of cytokine models in predicting SLE activity
Source: Arthritis Res Ther. 2019 Dec 16;21:287. doi: 10.1186/s13075-019-2029-1 (PMC6915901; doi:10.1186/s13075-019-2029-1)
Supplement: Supplementary file 3 — Additional file 3: Table S3. The correlation between cytokines and medications in all SLE patients. [file 13075_2019_2029_MOESM3_ESM.docx]

**Table S3. The correlation between cytokines and medications in all SLE patients**

| **Biomarkers** | | **Pred**  **(dose)** | **HCQ**  **(dose)** | **AZA**  **(dose)** | **CYC**  **(dose)** | **CYA**  **(dose)** | **MMF**  **(dose)** | **MTX**  **(dose)** | **TAC**  **(dose)** |
| --- | --- | --- | --- | --- | --- | --- | --- | --- | --- |
| IFN-α | R | 0.256 | 0.062 | 0.010 | -0.141 | -0.758 | 0.206 | 0.109 | 0.104 |
|  | P | ns | ns | ns | ns | ns | ns | ns | ns |
| MCP-1 | R | 0.137 | -0.009 | 0.004 | 0.214 | -0.817 | 0.259 | 0.114 | -0.117 |
|  | P | ns | ns | ns | ns | ns | ns | ns | ns |
| IL-6 | R | 0.152 | 0.072 | 0.089 | 0.025 | -0.870 | 0.174 | 0.113 | 0.118 |
|  | p | ns | ns | ns | ns | ns | ns | ns | ns |
| IL-8 | R | **0.389** | 0.059 | -0.184 | -0.008 | -0.877 | 0.121 | 0.138 | 0.118 |
|  | P | ****** | ns | ns | ns | ns | ns | ns | ns |
| IL-18 | R | **0.347** | 0.136 | -0.178 | -0.120 | 0.493 | 0.190 | 0.120 | 0.077 |
|  | P | ***** | ns | ns | ns | ns | ns | ns | ns |

R = Correlation coefficient; *p<0.05, **p<0.01, ns = non-significant (Bonferroni correction)
