# Supplementary material for: Performance of cytokine models in predicting SLE activity
Source: Arthritis Res Ther. 2019 Dec 16;21:287. doi: 10.1186/s13075-019-2029-1 (PMC6915901; doi:10.1186/s13075-019-2029-1)
Supplement: Supplementary file 4 — Additional file 4: Table S4. The correlation between cytokines, age, and gender in SLE patients. [file 13075_2019_2029_MOESM4_ESM.docx]

**Table S4. The Correlation between cytokines, age, and gender in SLE patients**

| **Biomarkers** | | **Age** | **Gender** |
| --- | --- | --- | --- |
| **IL-1β** | **R** | 0.007 | 0.000 |
|  | **P** | 0.332 | 0.805 |
| **IFN-α** | **R** | **0.065** | 0.001 |
|  | **P** | **0.030** | 0.677 |
| **IFN-γ** | **R** | 0.023 | 0.001 |
|  | **P** | 0.079 | 0.704 |
| **TNF-α** | **R** | 0.005 | 0.009 |
|  | **P** | 0.388 | 0.260 |
| **MCP-1** | **R** | 0.007 | 0.000 |
|  | **P** | 0.326 | 0.877 |
| **IL-6** | **R** | **0.038** | 0.001 |
|  | **P** | **0.023** | 0.706 |
| **IL-8** | **R** | 0.009 | 0.000 |
|  | **p** | 0.271 | 0.818 |
| **IL-10** | **R** | 0.009 | 0.000 |
|  | **P** | 0.274 | 0.895 |
| **IL-12** | **R** | 0.000 | 0.000 |
|  | **P** | 0.865 | 0.834 |
| **IL-17** | **R** | 0.011 | 0.006 |
|  | **P** | 0.226 | 0.374 |
| **IL-18** | **R** | 0.021 | 0.009 |
|  | **P** | 0.091 | 0.256 |
| **IL-23** | **R** | 0.004 | 0.002 |
|  | **P** | 0.456 | 0.544 |
| **IL-33** | **R** | 0.004 | 0.001 |
|  | **P** | 0.466 | 0.711 |
