# Supplementary material for: Performance of cytokine models in predicting SLE activity
Source: Arthritis Res Ther. 2019 Dec 16;21:287. doi: 10.1186/s13075-019-2029-1 (PMC6915901; doi:10.1186/s13075-019-2029-1)
Supplement: Supplementary file 5 — Additional file 5: Table S5. Predictors of SLE disease activity by logistic regression analysis. [file 13075_2019_2029_MOESM5_ESM.docx]

**Table S5. Predictors of SLE disease activity by logistic regression analysis**

| **Biomarkers** | **Weeks** | **Model** | **Active SLE**  **(N=51)** | | **Active Renal**  **(N=31)** | | **Active Non-Renal**  **(N=20)** | |
| --- | --- | --- | --- | --- | --- | --- | --- | --- |
|  |  |  | **ORs**  **(95%CI)** | **P-value** | **ORs**  **(95%CI)** | **P-value** | **ORs**  **(95%CI)** | **P-value** |
| C3  (<0.9 g/l) | 4 | Bivariate | **2.25(1.22-4.12)** | **0.009** | **3.33(1.66-6.68)** | **0.001** | 0.76(0.35-1.61) | 0.474 |
|  |  | Multiple | 1.63(0.69-3.87) | 0.260 | **2.68(1.01-7.05)** | **0.046** | - | - |
|  | 8 | Bivariate | **2.10(1.23-3.57)** | **0.006** | **3.06(1.67-5.60)** | **0.000** | 0.74(0.38-1.42) | 0.369 |
|  |  | Multiple | 1.69(0.80-3.55) | 0.167 | **2.72(1.16-6.36)** | **0.021** | - | - |
| C4  (<0.2 g/l) | 4 | Bivariate | **2.04(1.12-3.74)** | **0.020** | **2.52(1.30-4.91)** | **0.006** | 0.90(0.42-1.90) | 0.787 |
|  |  | Multiple | 1.36(0.59-3.16) | 0.464 | 2.03(0.97-4.23) | 0.059 | - | - |
|  | 8 | Bivariate | **1.82(1.07-3.09)** | **0.025** | **2.20(1.23-3.91)** | **0.007** | 0.88(0.46-1.70) | 0.715 |
|  |  | Multiple | 1.11(0.54-2.29) | 0.772 | 1.71(0.91-3.21) | 0.095 | - | - |
| Anti-dsDNA  (>100.0 IU/ml) | 4 | Bivariate | **1.99(1.06-3.75)** | **0.032** | **2.45( 1.26-4.76)** | **0.008** | 0.84(0.38-1.87) | 0.676 |
|  |  | Multiple | 1.88(0.87-4.05) | 0.105 | 2.10(0.99-4.46) | 0.052 | - | - |
|  | 8 | Bivariate | **2.14(1.22-3.75)** | **0.007** | **2.77(1.55-4.95)** | **0.001** | 0.76(0.38-1.54) | 0.460 |
|  |  | Multiple | **1.95(1.06-3.62)** | **0.032** | **2.09(1.09-4.02)** | **0.026** | - | - |
| IFN-α  (>10.0 pg/ml) | 4 | Bivariate | 1.68(0.90-3.13) | 0.097 | **1.95(1.01-3.76)** | **0.046** | 0.90(0.41-1.97) | 0.804 |
|  |  | Multiple | - | - | 1.24(0.59-2.62) | 0.556 | - | - |
|  | 8 | Bivariate | **1.96(1.14-3.38)** | **0.015** | **1.93(1.10-3.41)** | **0.022** | 1.13(0.58-2.19) | 0.713 |
|  |  | Multiple | 1.68(0.96-2.96) | 0.068 | 1.31(0.69-2.50) | 0.403 | - | - |
| MCP-1  (>960.0 pg/ml) | 4 | Bivariate | **2.25(1.23-4.13)** | **0.008** | 1.72(0.90-3.30) | 0.098 | 1.71(0.80-3.67) | 0.162 |
|  |  | Multiple | 0.72(0.31-1.64) | 0.438 | - | - | - | - |
|  | 8 | Bivariate | 1.39(0.82-2.35) | 0.215 | 0.98(0.56-1.71) | 0.946 | 1.72(0.89-3.34) | 0.105 |
|  |  | Multiple | - | - | - | - | - | - |
| IL-6  (>6.2 pg/ml) | 4 | Bivariate | **3.75(1.74-8.10)** | **0.001** | **2.64(1.28-5.43)** | **0.008** | 1.69(0.74-3.85) | 0.207 |
|  |  | Multiple | **3.50(1.51-8.11)** | **0.003** | **2.22(1.07-5.13)** | **0.040** | - | - |
|  | 8 | Bivariate | **2.13(1.13-4.01)** | **0.018** | **1.85(1.05-3.45)** | **0.043** | 1.30(0.68-2.70) | 0.476 |
|  |  | Multiple | **1.89(1.03-3.82)** | **0.042** | 1.61(0.98-3.17) | 0.166 | - | - |
| IL-8  (>55.0 pg/ml) | 4 | Bivariate | **3.54(1.86-6.72)** | **0.000** | **2.26(1.17-4.35)** | **0.014** | **2.23(1.04-4.77)** | **0.038** |
|  |  | Multiple | **3.43(1.62-7.27)** | **0.001** | **1.79(1.02-3.83)** | **0.039** | **2.23(1.04-4.77)** | **0.038** |
|  | 8 | Bivariate | **1.98(1.16-3.95)** | **0.012** | 1.46(0.98-2.57) | 0.052 | **1.69(1.02-3.26)** | **0.040** |
|  |  | Multiple | 1.64(0.90-2.99) | 0.106 | - | - | **1.69(1.02-3.26)** | **0.040** |
| IL-18  (>50.0 pg/ml) | 4 | Bivariate | **5.86(3.02-11.36)** | **0.000** | **4.57(2.29-9.11)** | **0.000** | 1.86(0.87-3.97) | 0.106 |
|  |  | Multiple | **5.57(2.63-11.72)** | **0.000** | **4.08(1.73-9.64)** | **0.001** | - | - |
|  | 8 | Bivariate | **4.22(2.39-7.46)** | **0.000** | **3.83(2.13-6.90)** | **0.000** | 1.38(0.71-2.66) | 0.332 |
|  |  | Multiple | **3.68(1.96-6.94)** | **0.000** | **3.90(1.93-7.86)** | **0.000** | - | - |
