# Supplementary material for: Performance of cytokine models in predicting SLE activity
Source: Arthritis Res Ther. 2019 Dec 16;21:287. doi: 10.1186/s13075-019-2029-1 (PMC6915901; doi:10.1186/s13075-019-2029-1)
Supplement: Supplementary file 6 — Additional file 6: Figure S1. The correlation of biomarkers and modified SLEDAI in SLE patients. [file 13075_2019_2029_MOESM6_ESM.pdf]

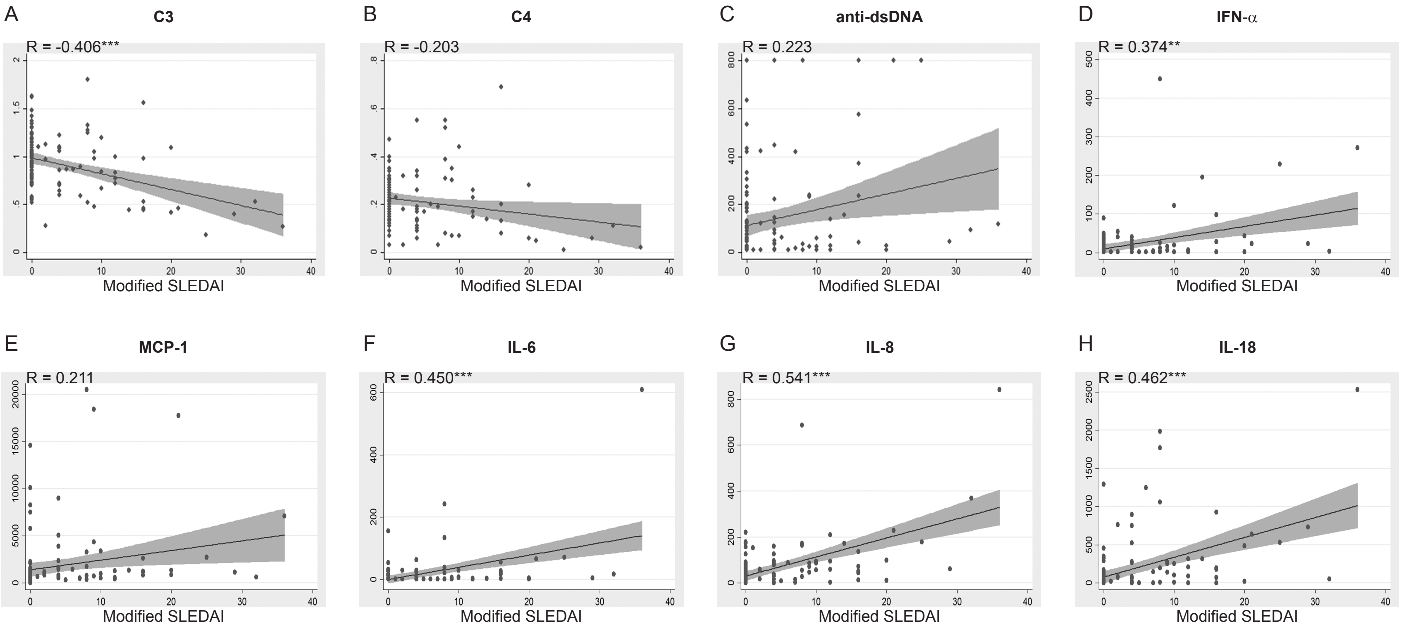

Fig. S1 Correlation of biomarkers and modified SLEDAI in SLE patients (N=124).
Pearson correlation coefficient (R) of C3 (A), C4 (B), anti-dsDNA (C), IFN-α (D), MCP-1 (E), IL-6 (F), IL-8 (G), and IL-18 (H) and modified SLEDAI scores were showed. The grey area is 95% confidence interval; $p<0.05$, $^{**}p<0.01$, and $^{***}p<0.001$ (Bonferroni correction).
