# Supplementary material for: Performance of cytokine models in predicting SLE activity
Source: Arthritis Res Ther. 2019 Dec 16;21:287. doi: 10.1186/s13075-019-2029-1 (PMC6915901; doi:10.1186/s13075-019-2029-1)
Supplement: Supplementary file 7 — Additional file 7: Figure S2. The correlation between each cytokine in all SLE patients. [file 13075_2019_2029_MOESM7_ESM.pdf]

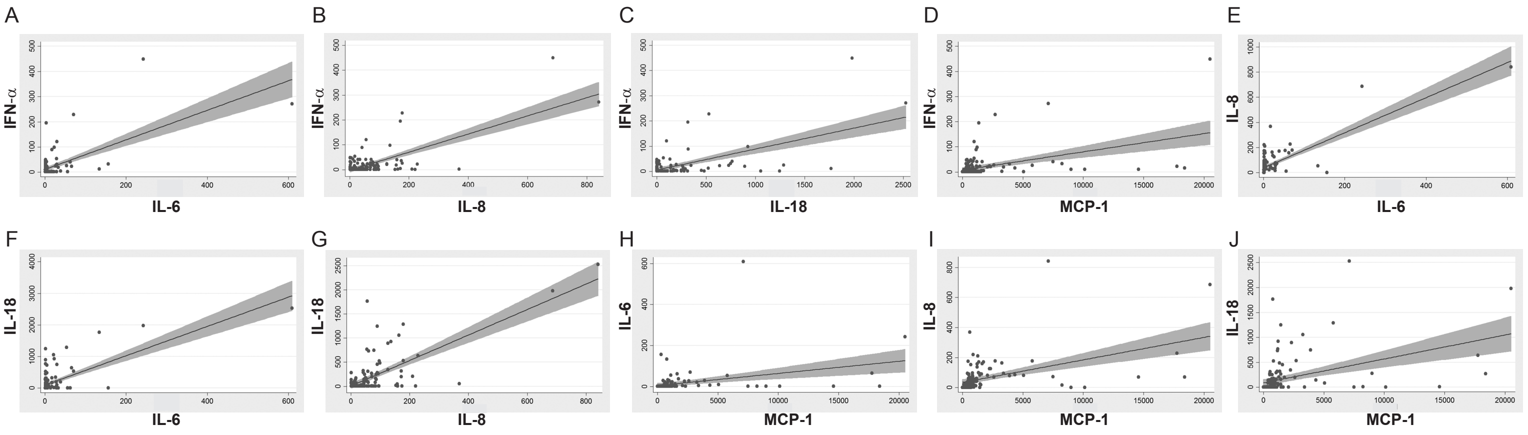

Fig. S2 The correlation between each cytokine in all SLE patients (N=124)
The correlation coefficient were shown in Table 3.
