# Supplementary material for: Performance of cytokine models in predicting SLE activity
Source: Arthritis Res Ther. 2019 Dec 16;21:287. doi: 10.1186/s13075-019-2029-1 (PMC6915901; doi:10.1186/s13075-019-2029-1)
Supplement: Supplementary file 8 — Additional file 8: Figure S3. Receiver operating characteristic (ROC) curve and area under the ROC curve in SLE patients. [file 13075_2019_2029_MOESM8_ESM.pdf]

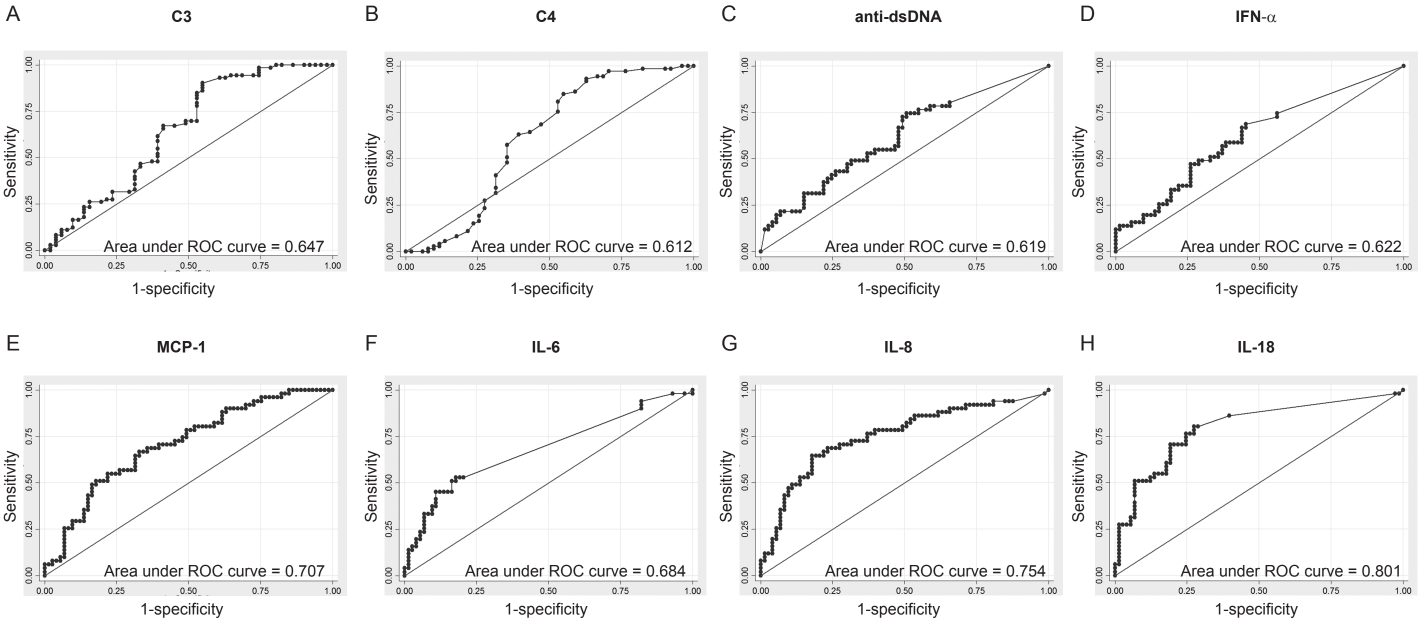

Fig. S3 Receiver operating characteristic (ROC) curve and area under ROC curve in SLE patients (N=124).
The following biomarkers of C3 (A), C4 (B), anti-dsDNA (C), IFN-α (D), MCP-1 (E), IL-6 (F), IL-8 (G), and IL-18 (H) were showed.
